# Resistant to Ablation, Responsive to Hydroquinidine: Precision Medicine Guides Recovery in MEPPC Syndrome Cardiomyopathy

**DOI:** 10.1002/joa3.70318

**Published:** 2026-05-08

**Authors:** Jonathan L. Ciofani, Hariharan Raju, Malcolm Anastasius

**Affiliations:** ^1^ Sydney Medical School The University of Sydney Sydney Australia; ^2^ Department of Cardiology Royal North Shore Hospital Sydney Australia; ^3^ Department of Epidemiology and Biostatistics, School of Public Health Imperial College London London UK; ^4^ Department of Cardiology Concord Repatriation General Hospital Sydney Australia; ^5^ Faculty of Medicine, Health and Human Sciences Macquarie University Sydney Australia

## Abstract

We report a case of MEPPC syndrome presenting with severe dilated cardiomyopathy due to a pathogenic *SCN5A* p.Arg814Trp variant. Genetic diagnosis enabled precision pharmacotherapy with hydroquinidine, which suppressed multifocal Purkinje‐origin ectopy resistant to catheter ablation and led to marked improvement in left ventricular function.

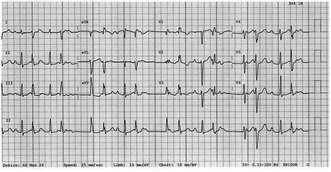

Multifocal Ectopic Purkinje‐related Premature Contractions (MEPPC) is a primary arrhythmogenic disorder resulting from *SCN5A* channelopathy. We report a case of MEPPC caused by the pathogenic *SCN5A* variant c.2440C>T (p.Arg814Trp) presenting with syncope and severe dilated cardiomyopathy, followed by genetic mutation specific pharmacologic treatment resulting in marked reduction in Purkinje system PVC burden and improvement in LV systolic function. Key learning points include: recognizing characteristic ECG findings in MEPPC; the limited role of ablation; and genetic testing enabling precision medicine pharmacotherapy.

A 44‐year‐old woman of Sri Lankan non‐consanguineous descent presented to hospital with syncope. Background included premature ventricular complexes during pregnancy 10 years earlier. At that time, cardiac magnetic resonance imaging (MRI) demonstrated normal biventricular size and function without late gadolinium enhancement (LGE), and no cardiology follow‐up was recommended. There was no family history of premature sudden cardiac death or cardiomyopathy. Initial 12‐lead electrocardiogram (ECG) and telemetry demonstrated apparent sinus rhythm with polymorphic premature ventricular contractions (PVCs) of both right and left bundle branch morphology, with sharp initial deflections, and relatively narrow QRS duration, consistent with fascicular or Purkinje origin. Subsequent ECGs demonstrated self‐terminating polymorphic ventricular tachycardia (Figure 1).

**FIGURE 1:**
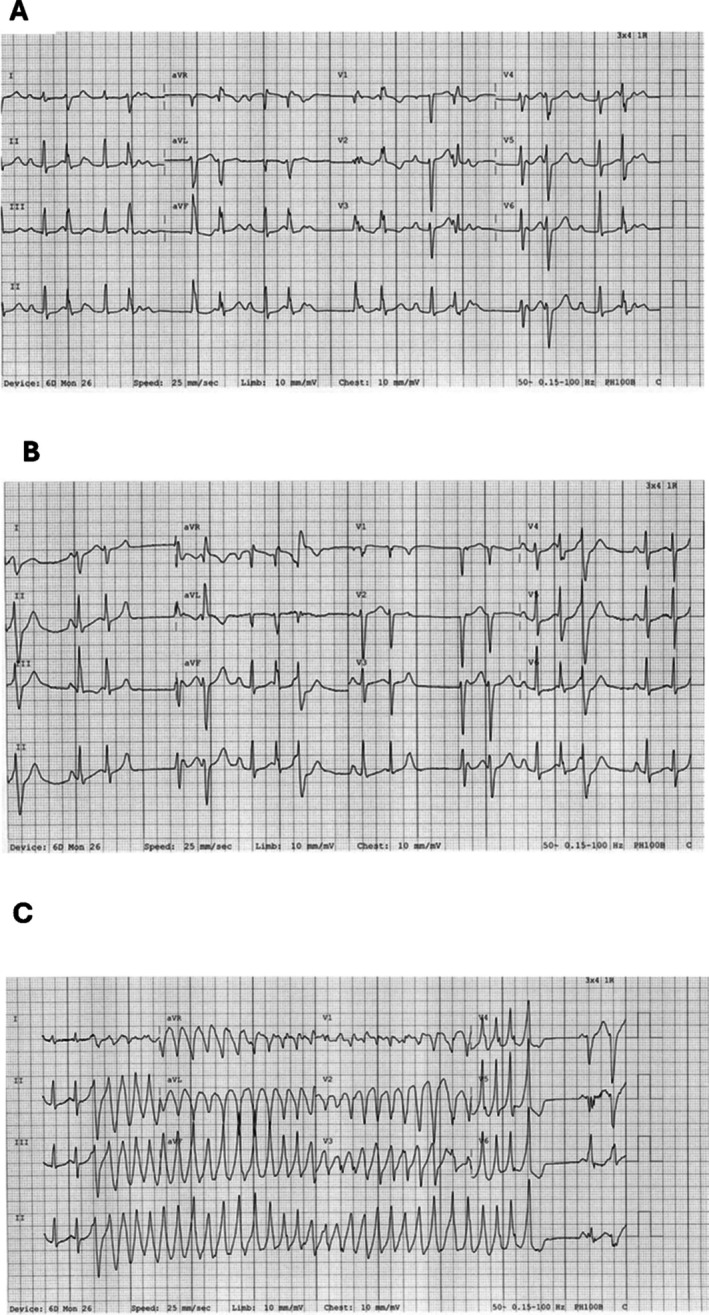
(A, B) Surface electrocardiogram demonstrating polymorphic premature ventricular contractions (PVCs) of both right and left bundle branch morphology, with sharp initial deflections, and relatively narrow QRS duration (120–150 ms). (C) Polymorphic ventricular tachycardia, with short coupled ectopic at onset.

Transthoracic echocardiography (TTE) revealed a severely dilated left ventricle (LV) with severe global systolic dysfunction and ejection fraction 25%–30%. Cardiac MRI was concordant, additionally demonstrating a small area of non‐ischemic mid‐wall to subepicardial LGE at the basal inferolateral wall, most likely representing scar due to prior myocarditis. Right ventricular size was at the upper limit of normal with mildly reduced systolic function. FDG‐PET showed no evidence of acute myocardial inflammatory conditions. Coronary angiography was normal.

Guideline directed heart failure therapy was initiated including bisoprolol, empagliflozin, and ramipril, with further intensification limited by hypotension. Given the presentation with syncope and polymorphic ventricular tachycardia, a dual‐chamber implantable cardioverter‐defibrillator (ICD) was implanted. Subsequent device interrogation identified paroxysmal atrial fibrillation, and anticoagulation was commenced.

Electrophysiology study was performed to further characterize the arrhythmia substrate. Baseline rhythm demonstrated an ectopic atrial rhythm originating from the left atrial septum, rather than sinus rhythm. Intracardiac recordings confirmed frequent PVCs of left anterior fascicular origin with Purkinje potentials preceding QRS complexes. There was evidence of conduction system disease with split His and HV 76 ms (Figure 2). High‐density electroanatomical mapping (CARTO 3) of the LV showed normal bipolar and unipolar voltage. Catheter ablation targeting left anterior fascicular PVCs resulted in temporary suppression, followed by recurrence from different fascicular sites. These findings also indicated that the MRI LGE inferolateral LV scar was not the source of PVCs.

**FIGURE 2:**
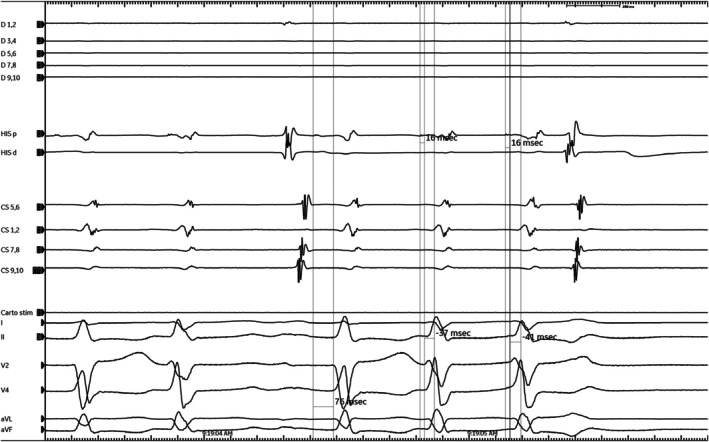
Electrophysiology study demonstrating prolonged HV interval (76 ms) and left anterior fascicular ectopy, notably with a short HV interval (16 ms) and His preceding the QRS.

Given the 12‐lead ECG and electrophysiologic findings suggested a channelopathy, genetic testing was performed which identified a heterozygous pathogenic gain‐of‐function variant in *SCN5A* (NM_000335.5:c.2440C>T, p.Arg814Trp) and a heterozygous variant of uncertain significance in *MYH7* (NM_000257.4:c.1562 T>C, p.Ile521Thr). The *SCN5A* p.Arg814Trp variant has been previously reported in patients with multifocal ectopic Purkinje‐related premature contractions (MEPPC) syndrome and affects the voltage‐sensing domain of the cardiac sodium channel.

Based on the clinical presentation of Purkinje‐related PVCs, dilated cardiomyopathy, and identification of a pathogenic *SCN5A* variant, a diagnosis of MEPPC syndrome was established. Treatment with hydroquinidine was commenced as an inpatient and titrated to a dose of 300 mg tds with serial ECGs demonstrating QTc < 500 ms and no proarrhythmic events. Six months after initiation, the 12‐lead ECG showed sinus rhythm without ventricular ectopy (Figure 3). After hydroquinidine commencement, the device‐detected ventricular ectopy burden also decreased from > 1000 to 80/h, and TTE demonstrated near normalization of LV systolic function (timeline provided in Figure 4). Family genetic counseling was provided, and cascade screening was recommended for first‐degree relatives.

**FIGURE 3:**
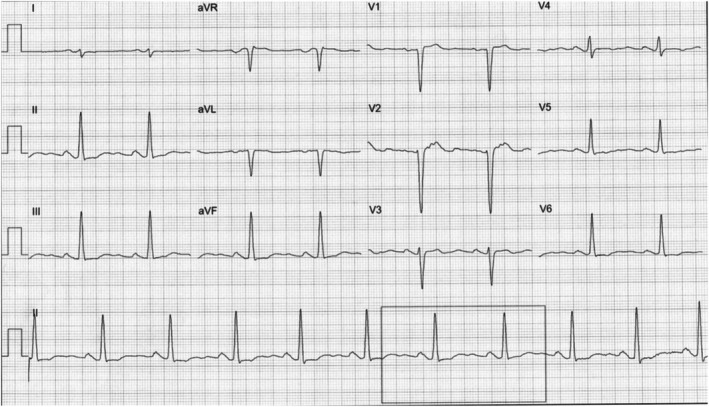
Surface electrocardiogram at 6 month follow up demonstrating sinus rhythm without ectopy.

**FIGURE 4:**
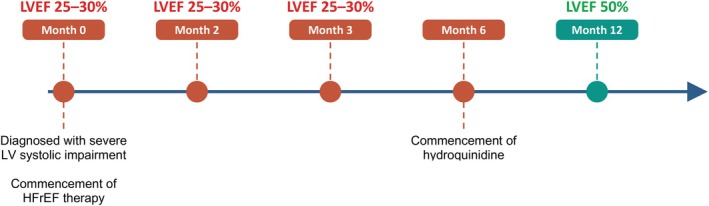
Timeline of left ventricular (LV) systolic function and therapy. Serial echocardiography demonstrated persistent severe LV systolic dysfunction at presentation, 2 months, and 3 months despite guideline‐directed therapy for heart failure with reduced ejection fraction (HFrEF). Following initiation of hydroquinidine after genetic confirmation of MEPPC syndrome, near‐normalization of LV systolic function was observed.

This case illustrates several key aspects of MEPPC syndrome. The SCN5A c.2440C>T mutation affects the S4 voltage‐sensing segment of Domain II, creating a gain‐of‐function effect [1]. This results in increased sodium influx and enhanced automaticity within the Purkinje system. The identification of this variant enabled a precision medicine approach, with institution of pharmacologic targeted sodium channel blockade resulting in suppression of PVCs and ventricular tachycardia from the fascicular‐Purkinje system and significant improvement in LV systolic function. Amiodarone has also demonstrated efficacy in select MEPPC cases, although Class I sodium channel blockers remain the preferred therapy given their direct mechanism of action on the underlying SCN5A gain‐of‐function defect.

Whilst our patient presented with severe dilated cardiomyopathy, this is not universal in MEPPC, with one series showing just 7 out of 21 patients with reduced LVEF [2]. Our patient had been lost to follow‐up for over 10 years, with prolonged high PVC burden likely contributing to LV dysfunction. Indeed, this case highlights why surveillance for apparently idiopathic ventricular ectopy is recommended. The improvement in LV function following hydroquinidine therapy demonstrates the reversible nature of MEPPC‐associated cardiomyopathy, aligning with previous reports of recovery following arrhythmia suppression [3]. Several mechanisms for cardiomyopathy development in MEPPC syndrome have been hypothesized; the reversibility observed in our patient and the history of previously normal cardiac MRI together favor arrhythmia‐mediated cardiomyopathy. Electroanatomical mapping confirmed the inferolateral LGE was not the arrhythmogenic substrate, and the MYH7 variant of uncertain significance does not alter this interpretation given the clearly pathogenic SCN5A variant and the near‐normalization of LV function following targeted sodium channel blockade.

Targeted medical therapy was essential for our patient's arrythmia suppression, with electrophysiology catheter ablation only achieving temporary suppression of fascicular‐Purkinje PVCs. Despite normal electroanatomical mapping and initial procedural success, ectopy recurred, originating from foci different from where ablation was attempted. This reflects the challenges in ablating multifocal arrhythmias, arising from an extensively distributed conduction system. Unlike unifocal fascicular tachycardia or re‐entry circuits, where targeted ablation can eliminate a discrete substrate, MEPPC syndrome involves diffuse Purkinje hyperexcitability that cannot be adequately addressed through focal ablation [4]. The concurrent ectopic atrial rhythm, detection of atrial fibrillation, prolonged HV interval and junctional escape beats additionally suggest *SCN5A* dysfunction may extend beyond the ventricular Purkinje system, consistent with prior reports of sinus node dysfunction and atrial arrhythmias [5].

This case underscores the importance of early diagnosis and genotype‐guided therapy in MEPPC syndrome before irreversible myocardial damage develops. Genetic evaluation should be considered in patients with multifocal fascicular ectopy and unexplained cardiomyopathy.

## Conflicts of Interest

The authors declare no conflicts of interest.

## Data Availability

The authors have nothing to report.
